# Hospital Food Service Strategies to Improve Food Intakes among Inpatients: A Systematic Review

**DOI:** 10.3390/nu13103649

**Published:** 2021-10-18

**Authors:** Noor Suzana Osman, Norazmir Md Nor, Mohd Shazali Md Sharif, Syahrul Bariah Abdul Hamid, Syafiqah Rahamat

**Affiliations:** 1 Faculty of Health Sciences, Puncak Alam Campus, Universiti Teknologi MARA, Puncak Alam 42300, Malaysia; noorsuzana@iium.edu.my (N.S.O.); syahrulbariah@uitm.edu.my (S.B.A.H.); 2 Kulliyyah of Allied Health Sciences, Kuantan Campus, International Islamic University Malaysia, Kuantan 25200, Malaysia; 3 Integrative Pharmacogenomics Institute, Puncak Alam Campus, Universiti Teknologi MARA, Puncak Alam 42300, Malaysia; 4 Faculty of Hotel and Tourism Management, Puncak Alam Campus, Universiti Teknologi MARA, Puncak Alam 42300, Malaysia; shazali@uitm.edu.my; 5 Faculty of Medicine and Health Sciences, Universiti Putra Malaysia, Seri Kembangan 43400, Malaysia; syafiqahrahamat@upm.edu.my

**Keywords:** foodservice, malnutrition, food intake, intervention, hospital

## Abstract

This review aims to identify hospital food service strategies to improve food consumption among hospitalized patients. A systematic search that met the inclusion and exclusion criteria was manually conducted through Web of Science and Scopus by an author, and the ambiguities were clarified by two senior authors. The quality assessment was separately conducted by two authors, and the ambiguities were clarified with all the involved authors. Qualitative synthesis was used to analyze and summarized the findings. A total of 2432 articles were identified by searching the databases, and 36 studies were included. The majority of the studies applied menu modifications and meal composition interventions (*n* = 12, 33.3%), or included the implementation of the new food service system (*n* = 8, 22.2%), protected mealtimes, mealtime assistance and environmental intervention (*n* = 7, 19.4%), and attractive meal presentation (*n* = 3, 8.3%). Previous studies that used multidisciplinary approaches reported a significant improvement in food intake, nutritional status, patient satisfaction and quality of life (*n* = 6, 16.7%). In conclusion, it is suggested that healthcare institutions consider applying one or more of the listed intervention strategies to enhance their foodservice operation in the future.

## 1. Introduction

Reduced food intake among hospitalized patients or inpatients is often associated with adverse health consequences such as malnutrition. Malnutrition is described as a lack or excess of nutrients, imbalance in macro- and micronutrient intakes, or both, resulting in irregular body structure, function, and clinical outcomes [1]. Malnutrition during hospitalization is a crucial problem; approximately 32% of patients are malnourished, and 23% eat less than 25% of the provided hospital food [2].

Malnutrition has several negative consequences, including a weakened immune system and slower wound healing, muscle wasting, longer hospital stays, increased treatment cost and a higher mortality rate [3]. A study showed that a lack of physical activity and/or a lower protein intake in patients due to the lower energy intake might result in muscle atrophy during a few days of hospitalization [4]. A low body mass index (BMI) at admission, concurrent illnesses and infections, a lack of food intake and quality, and male sex were significant factors influencing food intake and causing malnutrition among inpatients [5].

There are many factors associated with inadequate food intake among inpatients, such as lack of feeding aid, inability to provide daily healthy meals, and missing meals due to clinical investigations [1]. A previous statistic showed that about 58% of inpatients did not consume all the foods they were served [6]. According to the findings, factors related to food intake during hospitalization are related to both patients’ condition and the quality of the hospital food. Factors related to patients’ condition include physical characteristics, such as difficulties eating and swallowing. Psychosocial factors include being alone, neglected, stress and food beliefs, while examples of hospital food quality factors are unhygienic food and delayed mealtimes. These factors were reported to be significantly associated with increased food waste [7]. Moreover, nutritional impact symptoms include abdominal distention, dysphagia, diarrhoea, nausea, vomiting, lethargy, low appetite, being too sick or too tired to eat and poor dentition. The other conditions, such as interruptions during mealtime, not having food when a meal is missed and refusing to eat the ordered food were highly associated with inadequate food intake during hospitalization [8].

Identifying and managing malnutrition is essential because inappropriate nutritional support for inpatients with malnutrition leads to a higher transfer and mortality rate, longer hospital stay, and a lower discharge rate than well-nourished patients. It is suggested that future research should concentrate on the factors that contribute to insufficient food intake and the development of effective methods for reducing the risk of malnutrition in inpatients [9]. Additionally. the organization of food provision in hospital could harm patients’ food intake and nutritional status due to patients’ dissatisfaction with hospital meals, missed diagnoses due to inaccurate nutritional screenings and assessments, and the lack of training and hospital staff awareness [8,9,10]. Hence, it is essential to include a nutritional assessment as part of the patient’s clinical diagnoses. In addition, hospitals should develop systematic methods to prevent and treat malnutrition. These involve an interdisciplinary care team, such as a physician, dietitian, nurse, and pharmacist, working to develop a nutrition care plan, establish effective processes to diagnose malnourished patients and introduce comprehensive nutrition care plans [11].

Therefore, this systematic review aims to identify and integrate studies on hospital food service strategies to improve food intake among inpatients. This review considered the food service system, nutrition care plan, physical and environmental impact, and outcome strategies that help increase cost-effectiveness, optimize productivity, promote patients’ food intake, and improve nutritional care.

## 2. Materials and Methods

The Preferred Reporting Items for Systematic Reviews and Meta-Analyses (PRISMA) guidelines were used for the identification and evaluation of eligibility for the articles included in this systematic review [12] (Supplementary File S1). The systematic review was registered with the International Prospective Register for Systematic Reviews (PROSPERO) (CRD42021272357).

### 2.1. Search Strategy

Journals were searched using electronic databases from Web of Science and Scopus. At the first stage, the search strategy was a complete search string/query string and/or keywords, such as: “hospital foodservice”, “hospital”, “food service”, “catering”, “food quality”, “meal quality”, “patient satisfaction”, “food intake”, and “meal intake”. Boolean operators such as “AND” and “OR” were used where appropriate to combine the searches. Inclusion criteria such as publication years (2010 until 2019), document type (articles and conference), publication stage (final), source type (journals and conference proceedings), and language (English) were applied as search strategies during this stage. At the second stage, duplicate articles were identified, and the titles and abstracts were checked if they were relevant to the topic. Later, the filtered articles were further screened by reading individual manuscripts. Manuscripts that did not meet the requirements for inclusion were not considered.

### 2.2. Eligibility Criteria

After the screening process, the eligibility process was conducted according to the inclusion and exclusion criteria, which were selected based on the aims and objectives of the review paper. The study must be set up at the hospital food service area and conducted in any unit/ward in a hospital. The studies on relevant topics that were not conducted under the hospital food service process or dietetics department were excluded from the review process. The subjects involved in the study must be hospitalized patients who are 18 years old, and above, food service staff and/or other medical/healthcare professional staff such as nurses, doctors, medical assistance, and patients’ caregivers; these were also included as inclusion criteria. Published quantitative data papers in journals and conference proceedings were included in this study. The following parameters were set as inclusion criteria: patient food intake, nutritional status, patient satisfaction, plate waste, and quality of life. Studies on relevant topics that were not conducted under the hospital food service or dietetics department were excluded from the review process. Only complete manuscripts for journals and conference proceedings were included in this study.

### 2.3. Data Extraction and Management

Data extraction was carried out using a template created and verified specifically for this review. The data extracted from each study were the citation, aim or objectives, study population (study design, sample size, age group, and study duration), methodology (control and intervention groups), outcome parameters and findings. The first author manually performed the extraction (N.S.O.). The data extraction was then reviewed, and ambiguities were verbally clarified with the second and third senior authors (N.M.N. and M.S.M.S.). At the same time, quality assessment of the reviewed articles was separately conducted by the fourth and fifth authors (S.B.A.H. and S.R.). Later, the quality assessment was reviewed, and ambiguities were verbally clarified with all the authors involved in this review. The findings were summarized using qualitative synthesis by the first and forth authors, with the fifth author involved where necessary. The summarized results are categorized following the intervention strategies for hospitalized food services and improvements in food intake, nutritional status, or patient or overall hospital food service operation satisfaction.

### 2.4. Quality Assessment

The Quality Criteria Checklist for Primary Research (QCCPR) was used to determine each study’s quality, including criteria for assessing the study’s validity and bias [13]. The tool consists of four relevant questions that address applicability to practice. The research issue, sample population, sampling (bias and randomization), intervention or exposure, measurement results, statistical analysis, and interpretation of findings were among the ten validity questions [14]. The studies’ quality was rated as positive, neutral, or negative. A positive rating indicates that most aspects of the study met the validity criteria. In contrast, a neutral rating suggests that the study is not remarkably strong, according to the Academy of Nutrition and Dietetics’ QCCPR. A negative rating indicates that most of the study fails to meet the validity criteria.

### 2.5. Data Analysis and Synthesis

Meta-analysis was not possible due to the high heterogeneity across the studies included in this review. The authors decided to compare and summarize any statistical significance in the studies included in this review. The independent variables (the type of intervention study, such as food service system, menu modification, environment, and physical intervention) and dependent variables (food intake, food records, visual estimation tools, BMI, weight changes, patient satisfaction, quality of life, etc.) were evaluated by the first and fourth authors during the analysis, and all the findings were then compared and summarized in Table 1.

## 3. Results

### 3.1. Study Selection and Characteristics

**Figure 1:**
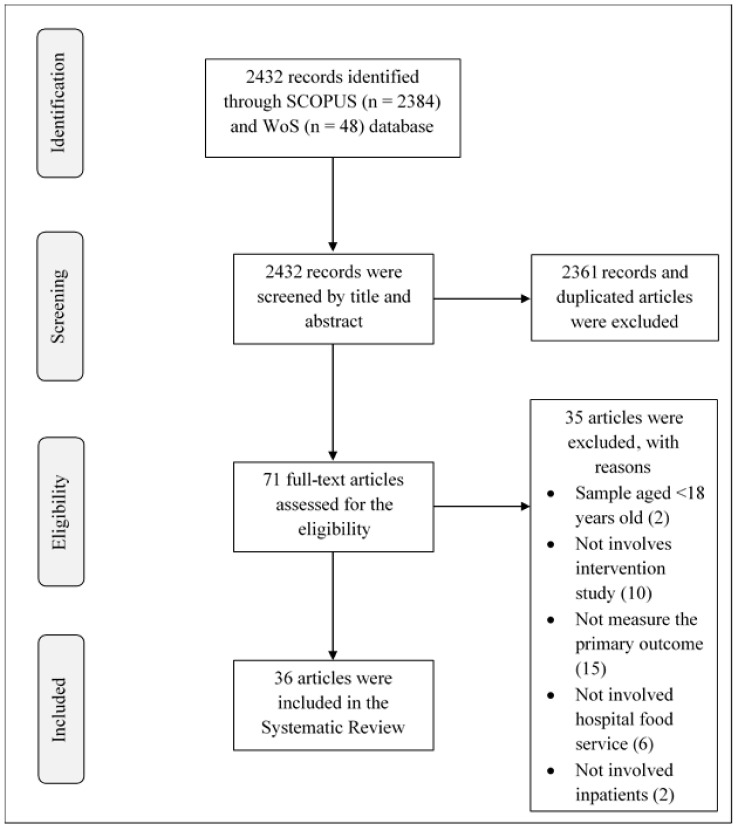
Flow chart for the literature search process.

The total number of articles found in the search database is shown in Figure 1. The literature search identified 2384 articles from Scopus and 48 articles from the Web of Science. Then, 2432 articles were screened by title and abstract, and 2361 irrelevant and duplicated articles were excluded. At the eligibility stage, 35 out of 71 full-text articles were excluded. The excluded articles did not meet the eligibility criteria; for example, the study population was less than 18 years old (*n* = 2), there was no intervention study (*n* = 10), no hospital food service (*n* = 6), food intake was not measured as the primary outcome (*n* = 15), or inpatients did not comprise the population sample (*n* = 2). Finally, 36 articles or studies were selected for this systematic review, as summarized in Table 2.

The characteristics of the selected studies are presented in Table 1. The selected studies were conducted worldwide between 2010 and 2019. Most of them were conducted in Australia [10,18,24,25,30,31,34,35,38,40,41,43,46], followed by Denmark [16,21,22,28,35,39,42,44], the Netherlands [15,23,26,36], Canada [47,48], Israel [17,45], and Spain [32], Hong Kong [29], Belgium [37], United Kingdom [27], Iceland [20], Switzerland [19], and India [33], respectively. Six articles were intervention studies [20,21,22,23,24,28], five were randomized control trial studies [15,16,17,18,19], four were pre–post studies [10,29,30,31], cohort studies [34,36,37,49] and pilot studies [35,39,40,41], three were observational studies [25,26,27], cross-sectional studies [32,33,38], and quasi-experimental studies [42,43,44]. Our selection additionally comprised non-randomized control trial studies [45], mixed-method studies [46], prospective interrupted time-series studies [47], and case study approaches [48]. The age range of the study population was 18–93 years old, while the sample size varied from 23 to 4000 subjects.

### 3.2. Foodservice Intervention Strategies on Food Intake

Table 1 summarizes the effects of the foodservice interventions implemented for each study, including study design, type of intervention strategy, results and summarized findings. Five food service intervention strategies aiming to improve patients’ intake and nutritional status were identified based on the studies’ aims, methods, output measures, results, and findings.

#### 3.2.1. Foodservice System Intervention

Eight studies (*n* = 8, 22.2%) implemented a new food service system in their hospital to improve patients’ food intake, focused on the meal-ordering system, service styles, and meal delivery [23,25,26,30,32,34,37,43]. Most of the studies reported positive improvements in patients’ food intake and satisfaction. They had a better meal quality, meal experience, oral nutritional support and reduced food waste and cost [23,25,26,30,32,37,43]. In contrast, one study showed no difference between bistro style and pre-plated services in terms of (i) energy or protein consumption, (ii) inpatient satisfaction, and (iii) meal quality [34].

#### 3.2.2. Menu Modification and Meal Composition Intervention

Menu modification and meal composition interventions were used in several studies (*n* = 12, 33.3%) to enhance patients’ food consumption, nutritional status, quality of life, and food production costs. Most of the studies reported improvements in total energy and protein, nutritional status, patients’ satisfaction, quality of life, as well as reductions in labour and food production cost [15,22,24,33,35,41,44]. Two studies reported no significant difference in total energy intake in both groups [16,20]. One study reported no significant difference in food intake over time; however, fat intake was increased during the intervention period [47]. Another study identified a positive relationship between meal portion size and plate waste, and reported increased food waste in patients at nutritional risk during supper [35].

#### 3.2.3. Multidisciplinary Approaches Intervention

Six studies (*n* = 6, 16.7%) adopted multidisciplinary approaches as their primary intervention strategy to improve patients’ food intake. The studies recorded interdisciplinary approaches at the individual-, ward-, and organizational level, or a combination of these. All studies reported an increase in food and nutrient intake, with a high percentage of patients meeting energy requirements, and showing improved body weight, increased patient satisfaction and increased quality of life [16,19,21,28,31,48].

#### 3.2.4. Protected Mealtime, Mealtime Assistance, and Environment Intervention

Seven studies (*n* = 7, 19.4%) applied protected mealtimes, mealtime assistance, and environmental interventions as their intervention approach to improve patient food intake [10,18,27,38,40,42,46]. A study was performed to establish the patient-related variables and aspects of protected mealtimes that correlated with adult inpatients’ energy and protein intakes [10]. Two studies (*n* = 2) that used a protected mealtime program showed an improvement in protein and energy intake among inpatients [10,38]. In contrast, one study reported no energy and protein intake changes in control and intervention groups, as well as a deficit in energy intake in the intervention group [18]. Two studies (*n* = 2) reported that mealtime assistance in the form of between-meal snacks served by the food caregiver led to an improvement in energy and protein intake, as it encouraged and motivated patients to eat [42,46]. One study implemented a mealtime environment indicative of patients’ preferences to have their meals in the dining room, based on the improvements seen in their energy and protein intake [40]. In contrast, another study reported a decrease in protein intake after the intervention, even though the mealtime environment was improved [27].

Randomized control-trial (RCT); intervention group (IG); historical intervention group (HIG); quality of life (QOL); not applicable (NA); oral nutritional supplements group (ONS); nutritional therapy group (NFT); good nutritional practice (GNP); bedside electronic meal ordering system (BMOS); paper menus (PMs); traditional foodservice model (TM); room service foodservice model (RS); protected mealtime program (PMP); traditional kitchen (TK); chilled kitchen (CK); dietary intake monitoring system (DIMS); traditional meal service (TMS); FoodforCare meal service (FfC); Meals on Wheels (MOW); historical control group (CG); malnutrition screening tool (MST); body mass index (BMI); hand grip strength (HGS); nutrition assistant (NA); Food Management System software (FMS); ready-to-use commercial modified texture food products (rMTF); commercial bulk modified-texture food products (cMTF); Mini Nutritional-Assessments (MNA).

#### 3.2.5. Meal Presentation Intervention

Of the 36 included studies, only three studies (*n* = 3, 8.3%) used meal presentation as their intervention strategy to improve the patients’ food intake [17,36,45]. All the studies reported improved food intake and satisfaction, as well as reductions in the intervention group’s food cost and readmission rate. The differences in protein intake per mealtime between the traditional three-meals-a-day food service (TMS) and a novel six-times-a-day food service, FoodforCare (FfC), which included protein-rich food products, was compared in a study that reported that the intervention group had a higher protein intake at all mealtimes except dinner [36]. However, the highest protein intake was recorded at dinner by both food services. Another study reported that loss-of-appetite patients who received meals with an improved display had a significantly higher food intake as compared to those who received a standard meal [45]. Patients in the orange napkin group consumed more hospital-provided food than those in the white napkin group [17]. The intervention group’s patients were also slightly more satisfied with the hospital’s food service.

### 3.3. Outcome Measures

#### 3.3.1. Food Intake

Food records, food weight, and visual estimation methods were identified as tools to determine the inpatient food intake in all studies included in this review. Nine out of 36 studies (25%) applied food records and showed a positive outcome in their studies [15,21,22,23,24,26,28,44,48]. Four of the nine studies (*n* = 4) used validated hospital foodservice management systems developed by the respective hospitals to determine the food intake of the patients [15,22,26,44]. Two studies used the traditional food intake record method, which involved a 24-h dietary recall interview and food record in nursing flow sheets, as the standard food intake monitoring method [21,48]. Nine studies (25%) used a scale to weigh meals prior to and after mealtime [19,27,29,32,36,37,41,42,46]. Additionally, 14 studies (38.8%) used visual estimation to record the portion size of the meal that was consumed [10,17,18,20,25,30,31,34,35,39,43,45,47,49]. Eleven studies recorded the portions of consumed food in exact percentages, while three studies used validated photographic software programs. Only two studies (6%) reported using both food record and visual estimation methods to verify patients’ dietary records in their studies [16,33].

#### 3.3.2. Patient Satisfaction

Patient satisfaction was assessed in ten of the 36 studies (27.8%), with most studies using validated questionnaires [17,24,26,28,30,33,34,35,41,43]. Two of them used the Acute Hospital Foodservice Patient Satisfaction Questionnaire (AHFPSQ), which was adapted from a previous study by Capra et al. [50]. The scores were given according to four domains: meal service, food quality, physical environment, staffing and service. The remaining studies used the validated patient satisfaction survey developed by their respective hospitals [17,24,38,40,44]. One study used the Nutrition Related Quality of Life Questionnaire to score six clusters on a scale from 1 to 6 (general health, food resource, food quality, service, and autonomy) [26]. One study used The Meal Assessment Tool to measure meal flavour, taste, appearance, and quality. In contrast, the Meal Quality Audit Tool was used to assess the sensory properties and temperature of the meal by the dietitian observers [34]. Only one study (10%) used a semi-structured interview guide to evaluate satisfaction, hosting intervention, and dining setting [28].

#### 3.3.3. Nutritional Status

The improvement in nutritional status was evaluated in eight studies (22.2%) in which body-weight changes were determined using a normal seated or standing weighing scale [18,26,30,39,41,43,47,48]. Handgrip strength was measured using hand dynamometers, as recorded in four studies (50%) [17,25,34,45], while the risk of malnutrition was evaluated in four studies (50%) using different validated malnutrition screening instruments [18,26,39,47], such as estimated ideal body weight formula, Subjective Global Assessment, Multi Universal Screening Tool (MUST, and Nutritional risk score-2002 (NRS-2002).

### 3.4. Quality Rating Studies

The overall quality of 27 selected studies was graded as positive by the Academy of Nutrition and Dietetics’ quality rating checklist [14,16,17,18,19,21,22,23,24,25,26,27,30,31,33,34,35,36,37,38,40,41,42,43,44,45,46], and nine selected studies were rated as neutral (Table 2) [10,15,20,28,29,32,39,47,49]. All studies clearly stated the research question and intervention descriptions. Thirty-four of the included studies used validated methods [10,14,15,16,17,18,19,21,22,23,24,25,26,27,29,30,31,32,33,34,35,36,37,38,39,40,41,42,43,44,45,46,47,49], while 35 studies reported the statistical analysis that was appropriate for the study and outcome indicators used, except for one study that did not report the appropriate analysis used in the study [47]. Most of the studies stated that participants’ selection was free from bias, except for two studies that unclearly noted the risk of bias [29,39]. In contrast, one study reported that participants’ risk of bias was not available [47]. Twenty-nine studies reported that the study groups were comparable [14,15,16,17,18,19,20,21,22,23,24,25,26,27,28,30,35,36,37,38,39,41,42,43,45,46,47]. The remaining six studies did not report this [10,29,31,32,33,48], with one study not clearly stating the study groups’ comparability [34]. Out of the 36 included studies, only eight studies reported that blinding was used to prevent the introduction of bias [14,16,17,20,26,27,38,47], while two studies did not identify or discuss the biases and study limitations [14,25]. All studies were reviewed, regardless of their quality rating or the reported intakes, to provide general explanations of the outcomes and potential study recommendations.

## 4. Discussion

Many factors are associated with malnutrition among inpatients. One of them is a decline in food consumption because of an illness-induced loss of appetite. A study in 56 countries showed that inpatients had inadequate food intake, which was significantly associated with reduced food intake [51]. Other significant factors are surgical procedures, concurrent illnesses and infection, low BMI upon admission, dissatisfaction with food quality, gastrointestinal symptoms, and inability to chew and swallow [5]. Regardless of age, gender, marital status, employment status, or diagnosis, a high prevalence of malnutrition among inpatients was associated with a longer hospital stay [52,53].

Nutritional intervention and strategies have significantly improved patients’ food intake, satisfaction, nutritional status, and quality of life, and reduced food waste and cost [23,25,26,30,33,37,44]. A new food service system was implemented using current technology that focused on the meal-ordering system, service styles, and meal delivery. For example, the use of electronic menus (E-menus) as an alternative approach to the meal-ordering system was an effective way to obtain information about the food, contributing to greater satisfaction among inpatients [54]. The bedside meal-ordering system showed improved food intake and patient satisfaction compared to traditional paper menu systems [25,43]. Assistance and guidance during meal orders can increase the suitability and consistency of orders, and monitor the nutritional status of patients. The meal-ordering system also helps determine patients that are at risk of malnutrition. It indirectly improves clinical outcomes where dietary education is needed [55]. Regardless of the use of new technology in the meal-ordering system, simple interventions such as verbal prompts for meal-ordering have proven to be a helpful tool to improve food consumption among patients during hospitalization [23].

Room service is now trending in many hospital food service operations. Room service increases patient satisfaction and food intake, while reducing food waste and cost [26,30]. Meal delivery systems play an essential role in monitoring and assessing patients’ food intake. Inpatients preferred the trolley system over the pre-plated meal system because the temperature was more controlled [56,57,58]. However, one study compared Bistro-style meals and pre-plated services and reported no significant differences in the patients’ food intake, satisfaction, and meal quality [34]. In a previous study comparing the same meal distribution system between prison and hospital food service, the delivery and service system were much less consistent (delay and disruption) in hospitals than in prison due to poor communication and the demands of medical professionals [59].

It is crucial to ensure patients’ total energy and protein intake meets the recommended requirements of the British Dietetic Association’s (BDA) and Nutrition and Hydration Digest Standard [60]. Most of the studies implemented menu modifications and composition interventions, such as energy- and protein-enriched meals or snacks, added condiments to the menu, and provided oral nutritional supplements with a combination of high-protein and high-energy snacks to the patient when promoting food intake [15,22,24,29,35,44]. It is suggested that total energy and protein requirements can be met by offering more energy-dense menu choices and optimizing the provision of hospital, snack, and oral nutritional supplements, as clinically recommended [61]. The patient-centered foodservice model is suggested to result in increased food intakes and improved nutritional status, as well as increases in patient satisfaction and quality of life, and reduced food costs [33]. The patient-centered model definition, in theory, benefits patients by improving communication, providing effective intervention, increasing satisfaction, and obtaining patient-reported outcomes [62].

This review also discovered that multidisciplinary approaches are one of the main intervention strategies to improve patients’ food intake. This interdisciplinary approach refers to active teamwork among the various healthcare team members to develop and deliver optimal care plans for inpatients [63]. It is a fundamental strategy to enhance the quality of food intake and patient wellbeing, decrease hospital stays, reduce costs, and support better health outcomes [64]. Multidisciplinary approaches to nutritional supervision are highlighted and indicated, regardless of whether they are individual, ward-based or organizational approaches, or a combination of the three. These have been reported to improve the patients’ food intake, nutritional status, satisfaction, and quality of life [16,19,21,28,31,48]. Nutrition interventions to tackle malnutrition are a low-risk, cost-effective approach to improving the quality of patient care; however, they require interdisciplinary collaboration. All healthcare team members (including dietitians, nurses, and physicians) are encouraged to communicate openly across disciplines and recognize the critical role of nutrition care in improving patient outcomes [65].

Protected mealtimes, mealtime environment, and mealtime assistance have been proven to be successful interventions to improve overall patients’ food intake. However, the effectiveness of protected mealtimes initiatives in increasing patients’ food intake has yet to be proven. Palmer and Huxtable [10] found many aspects of protected mealtimes to be linked to inpatient food intakes, including the introduction of mealtime volunteers and assistance and a proper mealtime atmosphere, which included conditions such as time and position during mealtimes. The same finding was revealed: food intake among elderly patients improved in the presence of meal assistants [66]. Markovski et al. [40] suggested that the dining room environment may positively impact food intake and enjoyment, potentially improving weight gain and nutritional status among elderly patients.

Furthermore, another study demonstrated that mealtime volunteers can improve mealtime treatment for adult patients or residents in institutional settings [67]. However, little well-designed research is available on mealtime volunteers or feeding assistance. By removing obstacles and creating an environment of support and personal attention during hospital mealtimes, feeding assistance is an essential technique for increasing elderly patients’ food intake [46]. Although the patients may experience various side effects and discomforts resulting from their illness, they still improved their food intake. Lindman et al. [42] also proposed that educated and trained food caregivers or assistants played a vital role in multi-professional nutritional management.

In contrast, Hickson et al. [27] reported that the protected mealtimes program in inpatients did not improve nutritional intakes, noting the energy deficit as a non-significant improvement. Another study by Porter et al. [18] also showed a limited improvement in food intake after implementation of the Protected Mealtime program. System-level nutrition intervention could increase food intake among patients at risk of malnutrition through fortified meals, mid-meals and mealtime assistance [38]. Previous studies reported that protein-supplemented hospital food substantially affected total protein intake and weight-adjusted energy intake among nutritionally vulnerable patients [68].

Furthermore, the meal presentation for cancer patients was also associated with higher plate wastage [69]. Food garnishes and attractive presentation encourage patients to try the food despite low appetites after treatment. Previous studies showed that patient satisfaction with hospital meals appeared to be strongly influenced by food variety, taste, presentation, flavour, and preparation [70,71,72,73]. Thus, a broader menu, high-quality taste, specific ingredient details, and improved mealtime, delivery, and food presentation will improve patient satisfaction with hospital foodservices [71,72]. Navarro et al. [45] found that enhanced meal presentation increases food consumption and patient satisfaction and decreases food costs and readmission rates. Research conducted by the same researchers, Navarro et al. [17], to compare the use of orange (experimental) and white (control) napkins on the inpatients’ meal intake showed improved patient satisfaction with hospital food service and increased food intake among patients with an orange napkin.

Moreover, implementing high-frequency food services containing protein-rich meals and attractive meal presentation led to improved protein intake at mealtimes during the day [31]. A recent study was conducted by Donnelly et al. [74] to compare the efficacy of blue versus white dishware in increasing food consumption and mitigating eating challenges among dementia residents. This systematic review concluded that the factors affecting food intake among residents living with dementia were complex. A simple intervention was insufficient to improve their dietary intake.

## 5. Limitations of Study

The key strength of this systematic analysis is the use of strict inclusion criteria, which ensures that appropriate intervention methods are chosen for hospital food services to increase patients’ food intake and nutritional status. Studies that were not conducted in healthcare settings were omitted because they did not measure the primary outcome and did not include inpatients. When evaluating the results of this systematic review, some limitations should be considered. This study used Clarivate Analytics’ Web of Science and Scopus databases as keyword-searching engines. Most of these databases, such as PubMed, Google Scholar, and Cochrane Library, were practical and offered various search facilities. However, Scopus covers a more comprehensive journal range and has a greater citation analysis capability than Web of Science. By comparison, the Web of Science features more attractive graphics and a more comprehensive overview of citations than the Scopus database [75]. Another constraint of this systematic analysis is that clinical heterogeneity was not considered. Heterogeneity is defined in a systematic review as any variation between studies, while clinical heterogeneity is defined as variation among the participants, treatments, and outcomes studied [76]. Although assessing clinical heterogeneity is relevant and should be considered in this systematic review, the authors have limited access to guidance in the processes of selecting potential effects and measuring modifiers. Additionally, variability is not always precisely quantified due to the imprecise definitions of intervention procedures, populations, and outcomes [77].

## 6. Conclusions

This review looks at evidence-based intervention strategies for hospital food service operators to improve patients’ food intake, satisfaction, nutritional status, and quality of life. Five intervention strategies were identified: implementing a new food service system, menu modification, multidisciplinary approaches in nutrition care, protected mealtime intervention programs, and attractive meal presentation. Although the meal presentation intervention strategy is less used in current hospital food service practice, it was evidenced to improve patients’ dietary intake and satisfaction, as well as reduce food cost and readmission rates. Thus, this review suggests that healthcare institutions should consider applying one or more of these interventions to improve their food service operations in the future.

## Figures and Tables

**Table 1:** Summary of individual studies related to the impact of hospital food service intervention strategies on inpatient’s food intake.

Author (s), (Year), Country	Study Design; Sample Size; Age Group; Study Duration	Types of Intervention Strategies	Outcome Parameters	Results	Summary of Findings
Beelen et al. (2018) [15], Netherlands	RCT; 147 patients (RCT: 67; control: 80); patients ≥65 years old; 7 months	Meal composition modification	Protein intake	Protein intake: RCT = 105.7 ± 34.2 g vs. Control = 88.2 ± 24.4 g (*p* < 0.01). More patients in RCT than control group reached a protein intake of 1.2 G/KG/D (79% vs. 47.5%).	High protein intake in the intervention group.
Munk et al. (2017) [16], Denmark	RCT; 91 patients (HIG = 41, and IG = 50); >18 years old; 8 months	Multidisciplinary approaches	Energy and protein intake Estimation of energy and protein requirement	>75% of energy requirement: IG = 92% vs. HIG = 76% (*p* = 0.04). >75% of protein requirement: IG = 90% vs. HIG = 66% (*p* = <0.01)	High mean energy and protein intake and the high number of patients reached >75% of energy and protein requirement in IG.
Navarro et al. (2019) [17], Israel	Intervention study randomized patients; 131 patients (white napkin: 65, orange napkin: 66); >18 years old; NA	Meal presentation	Food intake Patient satisfaction	IG consumed 17.6% more hospital-provided food than CG. IG significantly greater satisfaction with the hospital’s food service than CG.	Increase food intake and patient satisfaction in the intervention group.
Porter et al. (2017) [18], Australia	A prospective, stepped wedge cluster randomized controlled trial; 149 patients; ≥65 years old; 4 weeks	Protected mealtime	Energy and protein intake Nutritional status	Energy intake: IG (6479 ± 2486 kJ/day) vs. CG (6532 ± 2328 kJ/day), *p* = 0.88. Protein intake: IC (68.6 ± 26.0 g/day) vs. CG (67.0 ± 25.2 g/day), *p* = 0.86. Energy deficit: (coefficient [robust 95% CI], *p* value) of −1405 (−2354 to −457), *p* = 0.004	No significant difference in energy and protein intake for both groups. Significant finding in energy deficit.
Rüfenacht et al. (2010) [19], Switzerland	RCT; 36 patients (NT group = 18 vs. ONS group = 18); >18 years old; 10–15 days	Multidisciplinary approaches	Food intake QOL	Energy and protein intakes increased in both groups (*p* = 0.001). Energy intake that meets ER: NT (107%) vs. ONS (90%) Protein intake meets PR: NT (94%) vs. ONS (88%). QOL increased in NT (*p* = 0.016).	Increased QOL, energy and protein intake in both groups.
Ingadottir et al. (2015) [20], Iceland	Intervention study; 161 patients (2011 = 69, and 2013 = 92); ≥18 years old; 5 months	Menu modification	Energy and protein intake	Energy intake: IG (1293 ± 386 kcal/d) vs. CG (1096 ± 340 kcal/d), *p* = 0.001. Protein intake: IG (54.0 ± 17.8 g/d) vs. CG (49.1 ± 16.1 g/d), *p* = 0.085	Increased energy intake in the intervention group.
Holst et al. (2015) [21], Denmark	An observational multi-model intervention study; 545 patients (baseline = 287 patients, post intervention = 258 patients); >18 years old; 12 months from the baseline	Multidisciplinary approaches	Energy and protein intake Staff KAP regarding clinical nutrition GNP initiatives	Energy intake improved from 52% to 68% (*p* < 0.007). Protein intake from 33% to 52% (*p* < 0.001) (>75% of requirements). Intake of less than 50% of requirements decreased with 50%. Screening improved from 56% to 77% (*p* < 0.001). Nutrition plans from 21% to 56% (*p* < 0.0001). Monitoring food intake from 29% to 58% (*p* < 0.0001).	Improvement in energy and protein intake. Improvement of screening and monitoring the food intake process.
Beermann et al. (2016) [22], Denmark	Intervention study; 60 patients (baseline:32; follow-up:30); >18-year-old; 6 weeks	Menu modification	Energy intake Protein intake	Energy intake at breakfast: CG (14%) vs. IG (22%), *p* < 0.001. Protein intake breakfast: CG (14 g) vs. IG (20 g), *p* < 0.002. Total protein intake: CG (64%) vs. IG (77%), *p* = 0.05. Total energy intake: CG (76%) vs. IG (99%), *p* < 0.01.	Energy and protein intake were improved.
van der Zanden et al. (2015) [23], Netherlands	Intervention study; 208 patients (control = 63 vs. intervention = 145); ≥18 years old; 14 days of 4 consecutive weeks	Foodservice system	Protein intake Caloric content Ordering of the target product	Meal ordering: CG (6.5%) vs. IG (45.2%). Protein content: larger in IG > CG (*p* < 0.025)	High significant protein intake and content in the intervention group.
Campbell et al. (2013) [24], Australia	Intervention study; 98 patients (group 1 traditional: 33; group 2 MedPass: 32; group 3 mid-meal trolley: 33); ≥60 years old; 24 months	Menu and meal composition modification	Food intake Energy intake Protein intake Clinical measurements QOL Cost assessment Patient satisfaction	Weight changes (mean ± SD): traditional = 0.4 ± 3.8%, MedPass = 1.5 ± 5.8%, mid-meal = 1.0 ± 3.1% (*p* = 0.53) Energy intake and protein intake (% of requirement): traditional (107 ± 26, 128 ± 35%), MedPass (109 ± 28, 126 ± 38%), mid-meal = (85 ± 25, 88 ± 26%) (*p* = 0.003 and *p* < 0.001, respectively) QoL ratings (scale 0–100): MedPass (mean change, 12.4 ± 20.9), mid-meal (21.1 ± 19.7), traditional (1.5 ± 18.1) (*p* = 0.05). Patient satisfaction: sensory qualities (taste, look, temperature, size) and perceived benefit (improved health and recovery) was rated highest for mid-meal trolley (all *p* < 0.05).	Significantly increased food intake and patient satisfaction improved QoL and cost-effectiveness in the intervention groups. Energy and protein intake was achieved in both groups.
Barrington et al. (2018) [25], Australia	An observational point prevalence study; Oncology patients (BMOS: 105; PMs: 96); >18 years old; 18 months	Foodservice system	Dietary intake Plate waste Meal ordering Patient meal experience survey	Meal ordering: Energy = BMOS (8683 kJ day^−1^) vs. PM (6773 kJ day^−1^), *p* = 0.004); Protein = BMOS (97 g day^−1^) vs. PM (82 g day^−1^), *p* = 0.023. Food intake: Energy = BMOS (6457 kJ day^−1^) vs. PM (4805 kJ day^−1^), *p* < 0.001; Protein = BMOS (73 g day^−1^) vs. PM (58 g day^−1^), *p* < 0.001. Plate waste: BMOS (34.3 ± 4.9) vs. PM (35.3 ± 4.5), *p* = 0.75 Patient meal experience survey = significant increase in BMOS receiving ordered food (*p* < 0.001), able to choose their preferred food (*p* = 0.006) and able to assess nutritional information of the menu (*p* = 0.002) compared to the PM.	A significant increase in food intakes and meal experience improved upon access to nutritional information in the intervention group.
Doorduijn et al. (2016) [26], Netherlands	An observational prospective study; 337 patients (traditional meal system = 168, At Your Request^®^ = 169); ≥18 years old; 12 months	Foodservice system	Patient satisfaction Nutritional status Food choice and food intake	Patient satisfaction: increased after intervention from 7.5 to 8.1 (scale 1–10) and 124.5 to 135.9 point on a nutrition-related quality of life questionnaire (*p* < 0.05). Body weight: Traditional meal service (83.7 to 83.5 (0.2 ± 2.7) kg, *p* = 0.824), At Your Request^®^ (77.6 kg to 77.4 (0.2 ± 2.6) kg, *p* = 0.851) Handgrip strength: Traditional meal service (Day 1: 30.2 kg, End: 30.5 kg) vs. At Your Request^®^ (Day 1: 30.2 kg, End: 30.6 kg) MUST score: Improved in 18 patients in both groups. Protein intake (based on food records from patients on energy and protein enriched diet): Traditional meal service (*n* = 34, 0.91 g/kg) vs. At Your Request^®^ (*n* = 38, 0.84 g/kg).	Significantly higher intake of energy and protein, and patient satisfaction in the intervention group. MUST score improved in both groups.
Hickson et al. (2011) [27], United Kingdom	Direct observational study; 99 patients (baseline = 39, PM = 60); NA; baseline: June/July 2008, PM: Oct/Nov 2009	Protected mealtime	Mealtime experience Nutrient intake	Mealtime experience: Monitor using food/fluid charts (before PM (32%) vs. after PM (43%), *p* = 0.14); wash hands offer (before PM (30%) vs. after PM (40%), *p* = 0.03); served meals at uncluttered tables (before PM (54%) vs. after PM (64%), *p* = 0.04; experiencing mealtime interruptions (before PM (32%) vs. after PM (25%), *p* = 0.14). Energy intake: 1088 kJ vs. 837 kJ, *p* = 0.25 Protein intake: 14.0 g vs. 7.5 g, *p* = 0.25	Improvement in mealtime experience. There was a decrease in protein intake observed after the implementation of PM.
Holst et al. (2017) [28], Denmark	Interventional study: 67 patients (baseline = 30, follow up = 37); >18 years old; 3 months	Multidisciplinary approaches	Demographic information Energy and protein intake Patient-perceived quality Staff-perceived quality	Food intake: Energy intake: the overall group (67.6% vs. 40%; *p* = 0.036) vs. the Heart–Lung Surgery group (85.7 vs. 38.5; *p* = 0.036); Protein intake: the overall group (37.8% vs. 33.3%, *p* = 0.7037). Patient and staff-perceived quality: IG reported satisfaction regarding individualized food serving, nurse communication, and improved meal environments.	The food and energy intake, patient satisfaction on individualize meal serving and nurse communication, and meal environment were improved in the intervention group.
Chan et al. (2017) [29], Hong Kong	Pre-post design; 100 older patients (male: 49; female: 51); >65 years old; 3 months	Menu modification	Food intake	Food intake: IG (68%) vs. CG (57%). Increased intake of food, energy, protein, and sodium in IG by 8% (*p* < 0.05), 10% (*p* < 0.01), 9% (*p* < 0.01), and 53% (*p* < 0.01), in all patients, and by 13% (*p* < 0.01), 19% (*p* < 0.01), 17% (*p* < 0.01), and 67% (*p* < 0.01).	Increased intake of food, energy, protein, and sodium intake in lunch with condiments.
McCray et al. (2018) [30], Australia	Pre-post study design; 187 patients (TM = 84 and RS = 103 patients respectively); >18 years old; 1 month for each cohort	Foodservice system	Nutritional intake Plate waste Patient satisfaction Patient meal cost	Energy intake: TM (5513 kJ day^−1^) vs. RS (6379 kJ day^−1^), *p* = 0.020 Protein intake: TM (53 g day^−1^) vs. RS (74 g day^−1^), *p* < 0.001 Plate waste: TM (30%) vs. RS (17%), *p* < 0.001 Patient satisfaction: TM (75%) vs. RS (98%), *p* < 0.04 Food cost: decreased by 28% per annum with RS.	Significant increases in energy and protein intake, improved patient satisfaction, reduced plate waste and food cost in the intervention group.
Palmer and Huxtable (2015) [10], Australia	Pre-post study; 798 patients (Pre-PMP = 348 vs. Post-PMP = 450); >18 years old; 24 months	Protected mealtime and mealtime assistant	Food intake Aspects of protected mealtimes	Food intake: mean intake energy (1419 ± 614 kJ) and protein (15 ± 7 g); intakes associated with gender, age, season, stopping or refusing a meal, time until discharge and eating at dinner (*B* = − 829–222 kJ, *B* = − 8.8 to 2.2 g protein, *p* = 0.000–0.032); Intake in intervention group (*p* = 0.094–0.157); association of aspects of protected mealtimes with intake such as the need for mealtime assistance, introduction of mealtime volunteers, time to eat and appropriate positioning during mealtimes (*B* = 177–296 kJ, *B* = 0.07–3.9 g protein, *p* = 0.000–0.014, *R^2^* = 0.148–0.154). Protein intake in those requiring mealtime assistance was associated with mealtime volunteers and appropriate positioning (*B* = 4.1–4.4 g protein, *p* = 0.013–0.026, *R^2^* = 0.197).	The intake was associated with aspects of protected mealtimes, mealtime volunteers and appropriate positioning.
Roberts et al. (2019) [31], Australia	Observational, pre-post study; 207 patients (pre = 116 vs. post = 91); ≥18 years old; 2 months	Multidisciplinary approaches	Demographic data Food intakes Mealtime environment	Energy intake: Pre (4818 ± 2179 kJ) vs. Post (5384 ± 1865), *p* = 0.119 Protein intake: Pre (48 ± 24 g) vs. post (57 ± 22 g), *p* = 0.042 Mealtime interruption: Pre (111/423 meals) vs. Post (150/400 meals), *p* < 0.001. No. patients to receive their meal tray: Pre (76%) vs. Post (84%), *p* < 0.05	The number of patients with sufficient food consumption was doubled, and mean energy and protein intakes were significantly higher.
Calleja-fernández et al. (2017) [32], Spain	A cross-sectional, two-centre study; 201 patients (TK), 41 patients (CK); >18 years old; 18 months	Foodservice system	Energy intake Protein intake	Food intake: TK (median: 76.83%, IQR 45.76%) vs. CK (median: 83.43%, IQR 40.49%), *p* < 0.001 Energy intake: CK (1741.6 (SD 584.0) kcal) vs. TK (1481.7 kcal (SD 584.0) kcal) vs. TK (1481.7 kcal (SD 576.0) kcal); *p* = 0.014, after the statistical adjustment (1608.1 (SD 134.9) vs. 1466.8 kcal (SD 80.5) kcal; *p* = 0.243) Protein intake: CK (90.5 (SD 4.4) g) vs. TK (70.4 (SD 2.0) g); *p* < 0.001). after statistical adjustment (CK = 80.0 (SD 6.4) g vs. TK = 67.6 (SD 3.8) g; *p* = 0.032)	Higher energy and protein intake in the intervention group before the statistical adjustment.
Sathiaraj et al. (2019) [33], India	Cross-sectional analytical study; 160 patients (traditional foodservice = 60 vs. patient-centered foodservice = 100); >18 years old; 4 months	Menu modification	Nutritional intake Patient satisfaction	Energy intake: Patient-centered model: mean (SD) 1633.33 (158.11) kcal; Traditional foodservice model: mean (SD) 1501.67(171.22) kcal; *p* <0.001 Protein intake: Patient-centered model: mean (SD) 59.89 (10.897) kcal; Traditional foodservice model: mean (SD) 48.42 (10.794) g; *p* <0.001 In-hospital weight change: Patient-centered foodservice: mean (SD) 0.18 (0.99) kg; Traditional foodservice: mean (SD) −0.58 (1.25); *p* <0.001 Patient satisfaction: Quality of food (28.6 vs. 35.2%), timeliness of delivery (36.2 vs. 37.1%), flavour of food (21.9 vs. 37.1%), special/restricted diet explained (41 vs. 41.9%), and overall satisfaction (36.2 vs. 42.9%); *p* = 0.000	The mean of energy and protein intake, weight, and overall patient satisfaction in the intervention group was significantly increased.
Young et al. (2018) [34], Australia	Cross-sectional study; 30 patients (pre-plated *n* = 16; bistro style *n* =14); ≥65 years old; 4 weeks	Foodservice system	Dietary intake Patient satisfaction Meal quality	Energy intake: Bistro (2524 ± 927 kJ) vs. Pre-plate (2692 ± 857 kJ), *p* = 0.612 Protein intake: Bistro (29 ± 12 g) vs. Pre-plate (27 ± 11 g), *p* = 0.699 Patient satisfaction: appearance (preplated: 50%, Bistro: 46%), quality (preplated: 57%, bistro: 54%), staff demeanor (preplated: 100%, bistro: 92%) Meal quality: sensory properties (preplated: 4.2 ± 0.4, Bistro: 4.4 ± 0.7) and temperature accuracy (preplated: 3.1 ± 0.9, Bistro: 3.6 ± 1.3).	There is no difference in energy and protein intakes, patient satisfaction, or meal quality in both groups.
Ofei et al. (2015) [35], Denmark	Prospective observational cohort study; 71 patients (256 meals; lunch *n* = 142; supper *n* = 114); ≥18 years old; five weekdays	Menu and meal composition modification	Food intake Plate waste	Positive relationship between meal portion size and plate waste (*p* = 0.002) and increased food waste in patients at nutritional risk during supper (*p* = 0.001).	Increased the proportion of energy and protein consumption in both groups. There was a relationship between meal portion size and plate waste and increased food waste in patients at risk during supper.
Dijxhoorn et al. (2019) [36], Netherlands	A prospective cohort study; 637 subjects (TMS: 326, FfC: 311); ≥18 years old; TMS: 12 months, FfC: 12 months	Meal presentation	Protein intake per mealtime	Protein intake (g) at all mealtimes (*p* < 0.05) except for dinner (median (IQR) at breakfast: 17 (6.5–25.7) vs. 10 (3.8–17); 10:00 a.m.: 3.3 (0.3−5.3) vs. 1 (0−2.2); lunch: 17.6 (8.4−25.8) vs. 13 (7−19.4); 2:30 *p*.m.: 5.4 (0.8–7.5) vs. 0 (0–1.8); 7:00 p.m.: 1 (0–3.5) vs. 0 (0–1.7); 9:00 p.m.: 0 (0–0.1) vs. 0 (0–0)). Protein intake highest for both food services during dinner (20.9 g (8.4–24.1) vs. 20.5 g (10.5–27.8))	Protein intake higher in the intervention group except for dinner.
Goeminne et al. (2012) [37], Belgium	Prospective cohort trial; 189 patients (control = 83, MOW = 106); ≥18 years old; 2 months	Foodservice system	Food intake Food waste Food access and appreciation	Food intake: 236 g more in patients in the MOW group compared to controls (95% confidence interval: 163–308 g) Food waste: significantly less waste in the MOW group (*p* < 0.0001) Food access and appreciation: patients appreciated Meals on Wheels more than the old system in terms of choice (*p* = 0.048; OR 6.8; 95% CI (0.8–58)), hunger (*p* = 0.0012), food quality (*p* < 0.0001) and organization.	Food intake significantly increased for each meal, with reduced food waste, and greater ONS use in the MOW group. Patient noted increases in terms of choice, hunger, food quality and organization in MOW group.
Young et al. (2018) [38], Australia	Prospective cohort study; 320 patients (cohort 1 *n* = 129; cohort 2 *n* = 139; cohort 3 *n* = 52); ≥65 years old; 5 months for each cohort	Protected mealtime and mealtime assistant	Energy and protein intake Nutrition care process	Energy intake: cohort 1: 5073 kJ/d; cohort 2: 5403 kJ/d; cohort 3: 5989 kJ/d, *p* = 0.04 Protein intake: cohort 1: 48 g/d, cohort 2: 50 g/d, cohort 3: 57 g/d, *p* = 0.02	Energy and protein intakes were significantly improved between cohorts.
Munk et al. (2012) [39], Denmark	Historically controlled intervention pilot study; 40 patients; ≥ 18 years old; 10 weeks	Menu modification	Food intake	Energy intake: time gradient in energy intake (*p* = 0.0005, *r* = 0.53) Protein intake: 17.5% of the patients in the IG reached minimum *p* requirements (*p* = 0.17)	No significant difference in energy and protein intake in both groups. A significant time gradient was recorded in the energy intake.
Markovski et al. (2017) [40], Australia	A prospective observational pilot study; 34 patients; >65 years old; 3 months	Protected mealtime	Food intake MST	Food intake: patients consumed 20% more energy and protein when dining in a communal environment (*p* = 0.006 and 0.01, respectively) Patients with a BMI >22 (*p* = 0.01 and 0.01, respectively) and those with significant cognitive impairment (*p* = 0.001 and 0.007, respectively) ate 30% more protein and energy in the dining room, and those identified as at risk of malnutrition (MST ≥ 2) ate 42% more energy and 27% more protein in the dining room.	Higher energy and protein intakes and mealtime preferences among patients in the dining room.
Collins et al. (2017) [35], Australia	Parallel controlled pilot study; 122 geriatric patients; >65 years old; 4 months	Menu modification	Weight changes HGS Energy intake Protein intake Patient satisfaction	Weight changes: IG vs. CG (−0.55 (3.43) vs. 0.26 (3.33) %, *p* = 0.338) HGS change: IG vs. CG (mean (SD): 1.7 (5.1) versus 1.4 (5.8) kg, *p* = 0.798) Energy intake: IG vs. CG (mean (SD) 132 (38) vs. 105 (34) kJ/kg/day, *p* = 0.003). Protein intake: IG vs. C (mean (SD) 1.4 (0.6) vs. 1.1 (0.4) g protein/kg/day, *p* = 0.035) Patient satisfaction: food quality (*p* = 0.743), meal service (*p* = 0.559) or staffing and service (*p* = 0.816) scores, physical environment significantly higher among IG (*p* = 0.013).	Significant higher mean intake of energy and protein in the intervention group.
Farrer at al. (2015) [41], Australia	Pilot study; 66 patients (control group = 38, treatment group = 27); ≥18 years old; 2 weeks	Menu modification	Food intake Plate waste Patient satisfaction	Food intake: increased oral intake in the IG (*p* = 0.03) Plate waste: CG (median: 286 g) vs. IG (median: 160 g), *p* = 0.09 Patient satisfaction: no significant in both groups (*p* = 0.31)	Significantly increased food intake in the proportion of intervention group, but there was no significant change in all groups.
Lindman et al. (2013) [42], Denmark	Quasi-experimental; 87 patients (before = 42, after = 45); >18 years old; 1 year	Mealtime assistant	Food intake Nutritional requirement	Energy requirement: before-group (76.2% (CI 95% 64.6–87.9) vs. after-group (93.3% (CI 95% 82.3–104.3), *p* = 0.03. Energy intake: before-group 21 (51%) vs. after-group (30 (67%)), *p* = 0.145 Protein intake: before-group (16 (39%)) vs. after-group (16 (36%)), *p* = 0.74.	Higher energy intake in the intervention group. The patients were informed about their nutritional needs after the intervention.
Maunder et al. (2015) [43], Australia	The quasi-experimental pre-test post-test cohort study; 119 patients (PM = 54 patients, BMOS = 65 patients); ≥18 years old; 1 months for each phase	Foodservice system	Dietary intake Patient satisfaction NA role	Energy intake: PM vs. BMOS (6273 kJ vs. 8273 kJ), *p* < 0.05 Protein intake: PM vs. BMOS (66 g vs. 83 g), *p* < 0.05 Patient satisfaction: PM (84%) vs. BMOS (82%), *p* > 0.05. NA role: mean NA time with patients increased significantly from 0.33 to 0.35 min/patient/day (*p* < 0.05)	Most of the patients preferred the BMOS and mean daily energy and protein intakes were significantly increased in the intervention group.
Mortensen et al. (2019) [44], Denmark	A quasi-experimental design with a non-equivalent control group; 92 patients (46 before and 46 after the intervention; >18 years old; 11 months	Menu and meal composition modification	Energy and protein intake	Energy intake: increased from 74% to 109% (*p* < 0.00) of requirements. Protein intake: increased from 49% to 88% (*p* < 0.00) of requirements.	Increased total energy and protein intake from the requirements, including between meals.
Navarro et al. (2016) [45], Israel	The prospective open labeled, non-randomized controlled study; 206 patients (control = 101, experimental = 105); >18 years old; 3 weeks	Meal presentation	Food intake Food waste Readmission rate	Food intake: 9% significantly higher in the experimental group vs. control group (0.77 ± 0.25 vs. 0.58 ± 0.31) Food waste: starch Participants from the experimental group left on their plate less starch (experimental (0.19 ± 0.30) vs. control (0.52 ± 0.41), *p* < 0.05; main course (experimental (0.18 ± 0.31) vs. control (0.46 ±0.41), *p* < 0.05; vegetable (experimental (0.37 ± 0.36) vs. control (0.29 ± 0.35), *p* > 0.05. Readmission rate: control (31.2%) vs. experimental (13.5%), *p* < 0.02	There was significantly higher food intake, less food waste, improved food taste and decreased readmission rate in the intervention group.
Manning et al. (2012) [46], Australia	Mixed methods design; 23 patients; >65 years old; 3 months	Mealtime assistant	Food intake Grip strength MNA	Food intake: Energy and protein intakes increased significantly (396 kJ and 4.3 g, respectively) when volunteers were present. MNA: 52% at risk (MNA score between 17 and 23.5) and 35% malnourished (MNA score <17).	Energy and protein intake increased significantly during lunchtimes when volunteers were present.
Keller et al. (2012) [47], Canada	Prospective interrupted time-series study; 67 patients; ≥60 years old; 9 months	Menu and meal composition modification	Nutritional status Food intake Co-morbidity Oral supplements	Nutritional status: 74% patients achieved their goal weight at the end of the intervention period. Food intake: nonsignificant decrease in total grams of main-plate food consumed during the six-month intervention period when compared with the control period (*p* = 0.11).	Most of the patients in the intervention group achieved their weight goals. No significant difference in main-plate food intake. Higher fat intake in the intervention group.
Laur et al. (2019) [48], Canada	Case study approach; 4000 patients (Site A: 1127, Site B: 860, Site D: 988, Site E: 968); ≥18 years old; 18 months	Multidisciplinary approaches	Food intake Body weight	Food intake monitoring: Site A (Increased from 0% to 97%). Site E (increased from 0% to 61%). Site B (improved from 3% to 95%). Body weight monitoring: Site A (improved from 14% to 63%), Site D (improved from 11% to 49%).	Food intake and body weight improved through interdisciplinary team approaches and documentation.

**Table 2:** Quality assessment using the Quality Criteria Checklist for Primary Research of the 36 included studies in a systematic review of hospital food service.

Reference	Validity Items ^a^										Quality Rating
	1	2	3	4	5	6	7	8	9	10	
Beelen et al. (2018) [15]	+	+	+	+	+	+	+	+	-	-	Positive
Munk et al. (2017) [16]	+	+	-	-	U	+	+	+	+	NA	Neutral
Navarro et al. (2019) [17]	+	+	+	NA	+	+	+	+	+	NA	Positive
Porter et al. (2017) [18]	+	+	+	U	+	+	+	+	+	+	Positive
Rüfenacht et al. (2010) [19]	+	+	+	+	-	+	+	+	+	-	Positive
Ingadottir et al. (2015) [20]	+	+	+	-	-	+	+	+	+	+	Positive
Holst et al. (2015) [21]	+	+	+	+	-	+	+	+	+	U	Positive
Beermann et al. (2016) [22]	+	+	+	+	-	+	+	+	+	-	Positive
van der Zanden et al. (2015) [23]	+	+	+	NA	+	+	U	+	+	+	Neutral
Campbell et al. (2013) [24]	+	+	+	U	U	+	+	+	+	+	Positive
Barrington et al. (2018) [25]	+	+	+	-	-	+	+	+	+	+	Positive
Doorduijn et al. (2016) [26]	+	+	+	+	-	+	+	+	U	+	Positive
Hickson et al. (2011) [27]	+	+	+	+	+	+	+	+	+	+	Positive
Holst et al. (2017) [28]	+	+	+	+	+	+	+	+	+	U	Positive
Chan et al. (2017) [29]	+	+	+	-	-	+	U	+	+	-	Neutral
McCray et al. (2018) [30]	+	U	-	+	-	+	+	+	+	+	Neutral
Palmer and Huxtable (2015) [10]	+	+	+	+	U	+	+	+	+	+	Positive
Roberts et al. (2019) [31]	+	+	-	+	NA	+	+	+	+	+	Neutral
Calleja-fernández et al. (2017) [32]	+	U	-	+	-	+	+	+	+	+	Neutral
Sathiaraj et al. (2019) [33]	+	+	-	+	-	+	+	+	+	+	Neutral
Young et al. (2018) [34]	+	+	U	+	-	+	+	+	+	+	Neutral
Ofei et al. (2015) [35]	+	+	+	+	-	+	+	+	+	-	Positive
Dijxhoorn et al. (2019) [36]	+	+	-	+	-	+	+	+	+	+	Positive
Goeminne et al. (2012) [37]	+	+	+	-	-	+	+	+	+	-	Positive
Young et al. (2018) [38]	+	+	+	+	+	+	+	+	+	+	Positive
Munk et al. (2012) [39]	+	+	+	+	-	+	+	+	+	+	Positive
Markovski et al. (2017) [40]	+	+	+	+	-	+	+	+	+	+	Positive
Collins et al. (2017) [35]	+	+	+	+	-	+	+	+	+	+	Positive
Farrer et al. (2015) [41]	+	+	+	U	-	+	+	+	+	+	Positive
Lindman et al. (2013) [42]	+	+	+	+	-	+	+	+	+	+	Positive
Maunder et al. (2015) [43]	+	+	+	+	NA	+	+	+	+	+	Positive
Mortensen et al. (2019) [44]	+	+	+	+	NA	+	+	+	+	+	Positive
Navarro et al. (2016) [45]	+	+	+	NA	NA	+	+	+	+	+	Positive
Manning et al. (2012) [46]	+	+	+	+	NA	+	+	+	+	-	Positive
Keller et al. (2012) [47]	+	+	+	NA	+	+	+	+	+	NA	Positive
Laur et al. (2019) [48]	+	NA	-	-	-	+	+	U	+	+	Neutral

^a^ Study were rated on 10 items: 1 = Research question stated, 2 = Subject selection free from bias, 3 = Comparable study group, 4 = Method for withdrawals described 5 = Blinding used, 6 = Interventions describe, 7 = Outcomes stated and measurements valid and reliable, 8 = Appropriate statistical analysis, 9 = Appropriate conclusions and limitations described, and 10 = Funding and sponsorship free from bias. Shaded areas indicate that validity items must be satisfied for a positive quality rating. + = item present. - = item not present. NA = not applicable. U = unclear.

## Data Availability

Not applicable.

## Supplementary Materials

The following are available online at https://www.mdpi.com/article/10.3390/nu13103649/s1, Supplementary File S1: PRISMA 2020 Checklist.
